# Hemostatic function to regulate perioperative bleeding in patients undergoing spinal surgery: A prospective observational study

**DOI:** 10.1371/journal.pone.0179829

**Published:** 2017-06-16

**Authors:** Atsushi Kimura, Tsukasa Ohmori, Asuka Sakata, Teruaki Endo, Hirokazu Inoue, Satoshi Nishimura, Katsushi Takeshita

**Affiliations:** 1 Department of Orthopaedics, Jichi Medical University School of Medicine, Tochigi, Japan; 2 Department of Biochemistry, Jichi Medical University School of Medicine, Tochigi, Japan; 3 Division of Cell and Molecular Medicine, Center for Molecular Medicine, Jichi Medical University School of Medicine, Tochigi, Japan; International University of Health and Welfare School of Medicine, JAPAN

## Abstract

Although bleeding is a common complication of surgery, routine laboratory tests have been demonstrated to have a low ability to predict perioperative bleeding. Better understanding of hemostatic function during surgery would lead to identification of high-risk patients for bleeding. Here, we aimed to elucidate hemostatic mechanisms to determine perioperative bleeding. We prospectively enrolled 104 patients undergoing cervical spinal surgery without bleeding diathesis. Blood sampling was performed just before the operation. Volumes of perioperative blood loss were compared with the results of detailed laboratory tests assessing primary hemostasis, secondary hemostasis, and fibrinolysis. Platelet aggregations induced by several agonists correlated with each other, and only two latent factors determined inter-individual difference. Platelet aggregability independently determined perioperative bleeding. We also identified low levels of plasminogen-activator inhibitor-1 (PAI-1) and α2-plasmin inhibitor to be independent risk factors for intraoperative and postoperative bleeding, respectively. Most important independent factor to determine postoperative bleeding was body weight. Of note, obese patients with low levels of PAI-1 became high-risk patients for bleeding during surgery. Our data suggest that bleeding after surgical procedure may be influenced by inter-individual differences of hemostatic function including platelet function and fibrinolysis, even in the patients without bleeding diathesis.

## Introduction

Bleeding is a common complication of surgery, causing significant morbidity and mortality. Acute blood loss induces peripheral circulatory failure, which increases the risk of serious complications such as stroke, myocardial infarction, and pulmonary embolism [1, 2]. Furthermore, postoperative bleeding may cause symptomatic hematoma, leading to consequent neurological or blood flow deterioration that requires emergency reoperation [3]. The identification of bleeding patients before surgery would be desirable to evaluate unrecognized diseases and risk factors. However, routine preoperative laboratory tests are not recommended to categorize the risk, because of their poor predictive value, unnecessary costs, and potential delay of surgery [4, 5]. A simple screening questionnaire taking past history is the cornerstone of the preoperative assessment [6].

One reason why the laboratory measurements could not be applied to clinical practice is that factors to determine bleeding after injury are not fully comprehended. Better understanding of hemostatic mechanisms during surgery will lead to the development of reliable laboratory tests to identify the bleeding risk during surgery. In this study, we analyzed the association between perioperative bleeding and the measurement of several hemostatic markers, including platelet, coagulation, fibrinolysis, and vascular functions, in a prospective observational study to elucidate the mechanisms of hemostasis during surgery.

## Methods

### Patients and study protocol

The study was conducted in agreement with the Declaration of Helsinki. The institutional review board at Jichi Medical University approved the study protocols, and written informed consent was obtained from all participants. To eliminate the influence of routine prophylactic anticoagulation, we selected patients undergoing spine surgery. Because a consensus guideline for preventing venous thromboembolism does not exist [7] and venous thromboembolism is uncommon in spinal surgery [8], our institution has employed only mechanical thromboembolic prophylaxis after spine surgery. We prospectively enrolled 107 consecutive patients undergoing cervical laminoplasty for compressive cervical myelopathy from July 2010 to April 2013. Because three patients were excluded from the analysis (two because of inadequate blood sampling and one because of data omission), 104 patients were included in the analysis. We estimated the sample size required using a general formula for the correlation coefficient [9]. We set α = 0.05 and β = 0.20, and expected a correlation coefficient (*r*) = 0.30–0.35. Using the formula, at least 62–85 participants would be required for the study. Exclusion criteria were a platelet count of <10 × 10^7^/mL or >40 × 10^7^/mL; myeloproliferative disorders; malignancy; and previous history of unwarranted bleeding. Patient characteristics are shown in Table 1. A participant was deemed to have hypertension, diabetes mellitus, or rheumatoid arthritis if the participant was on medication for the comorbidity. Fourteen patients had received prophylactic antithrombotic medications (aspirin in seven patients, warfarin in two, aspirin and clopidogrel in two, clopidogrel in one, ticlopidine in one, and dabigatran in one). These oral antithrombotic medications were completely discontinued 7 days before surgery, but nine patients considered to be at high risk of recurrent thromboembolism were administered intravenous unfractionated heparin (10,000 U/day) until 6 h before the operation.

**Table 1:** Patient characteristics and preoperative laboratory parameters*.

Number of patients	104
Age (year)	65.6 ± 10.5
Male gender	65 (62.5%)
Weight (kg)	61.1 ± 13.9
BMI (kg/m^2^)	23.7 ± 3.7
Antithrombotic medication	14 (13.5%)
Comorbidity	
Hypertension	45 (43.3%)
Diabetes mellitus	22 (21.2%)
Rheumatoid arthritis	5 (4.8%)
Hemodialysis	6 (5.8%)
No. of enlarged laminae	
6 (C2-C7)	14 (13.5%)
5 (C3-C7)	80 (76.9%)
4 (C3-C6)	10 (9.6%)
Operation time (min)	185.4 ± 54.0
Intraoperative bleeding volume (ml)	99.8 ± 120.8
Postoperative bleeding volume (ml)	366.5 ± 147.9
White blood cell (10^9^/L)	6.3 ± 1.7
Red blood cell (10^10^/L)	418.5 ± 49.5
Hemoglobin (g/dL)	13.4 ± 1.6
Platelet count (10^10^/L)	22.0 ± 6.4
AST (U/L)	24.2 ± 14.7
ALT (U/L)	24.0 ± 19.3
Blood urea nitrogen (mg/dL)	16.0 ± 7.5
Creatinine (mg/dL)	1.0 ± 1.4
CRP (mg/dL)	0.2 ± 0.5
ESR (mm/hr)	17.5 ± 15.9
%PT (%)	98.5 ± 13.5
APTT (sec)	32.0 ± 5.9
PAI-1 (ng/ml)	26.7 ± 13.8
D-dimer (μg/ml)	1.0 ± 1.3
FDP (μg/ml)	4.9 ± 5.6
Soluble fibrin (μg/ml)	6.0 ± 3.6
E-selectin (ng/ml)	21.8 ± 10.6
Plasminogen (%)	93.3 ± 16.8
α2-PI (%)	87.6 ± 15.7
Antithrombin (%)	88.3 ± 16.5
PIC (μg/ml)	0.9 ± 0.3
Platelet coagulation (%)	
ADP-induced (2 μM)	47.0 ± 25.4
ADP-induced (5 μM)	64.1 ± 19.2
Collagen-induced (1 μg/ml)	61.9 ± 26.0
Collagen-induced (3 μg/ml)	78.7 ± 14.2
PAR-induced	50.8 ± 30.2

*Data are reported as number (%) or mean ± standard deviation. BMI, Body-mass index; AST, aspartate aminotransferase; ALT, alanine aminotransferase; CRP, C-reactive protein; ESR, erythrocyte sedimentation rate; %PT, %prothrombin time; APTT, activated thromboplastin time; PAI-1, plasminogen activator inhibitor-1; FDP, fibrin degradation products; α2-PI, α2-plasmin inhibitor; PIC, plasmin-α2-plasmin inhibitor complex; ADP, adenosine diphosphate; PAR, protease-activated receptor agonist.

### Blood sampling, platelet aggregation, and laboratory testing

A fasting venous blood sample was carefully collected via a 21-gauge needle into a syringe containing a 1:10 concentration of sodium citrate between 07:30 and 08:00 (just before the operation). Platelet aggregation measured as light transmission was performed using a PA-200 platelet aggregation analyzer (Kowa, Tokyo, Japan) [10]. Maximal light transmission (%) during 5 min of observation was applied as data for platelet aggregation. Plasma levels of total plasminogen activator inhibitor-1 (PAI-1) antigen, plasmin α2-plasmin inhibitor complex (PIC), D-dimer, plasminogen, α2-plasmin inhibitor (α2-PI), antithrombin (AT), soluble fibrin (SF), and E-selectin were assayed using an automated latex agglutination assay or chromogenic method (LPIA-NV7; LSI Medience, Tokyo, Japan). The intra- and inter-assay coefficients of each test were all <10%. The limit of PAI-1 detection is 10 ng/ml (reference value <50 ng/ml).

### Surgical procedure

The procedure for double-door laminoplasty has been described in detail elsewhere [11]. At the end of the procedure, a suction drain was placed deep to the fascia in all patients and maintained until the drainage rate decreased to less than 50 mL/day. Intraoperative blood loss was calculated from blood retrieved from wound suction and the estimated amount of blood in the gauze used intraoperatively. Postoperative bleeding was recorded as the total amount of drainage during the first 48 h.

### Statistical analysis

All statistical analyses were performed with SPSS Statistics for Windows version 17.0 (SPSS, Chicago, IL, USA) or JMP for Macintosh version 10.0 (SAS Institute, Cary, NC, USA). Associations between individual parameters were calculated using Pearson’s rank correlation tests. Categorical variables were given a dummy score, and correlations with volumes of blood loss were calculated. Multivariate linear regression analysis (stepwise forward approach) was performed to identify independent predictors. Factor analysis was employed to identify latent variables to explain the fluctuation of variables. All *P* values are two-sided; a *P* value of less than 0.05 was considered to be statistically significant.

## Results

### Patients’ characteristics and perioperative bleeding volume

The main purpose of this study was to investigate the mechanism of hemostasis in a traumatic bleeding such as an operation by measuring a variety of laboratory tests in a prospective cohort. One hundred and four patients were enrolled (65 male, 39 female; mean age 65.6 ± 10.5 years) (Table 1). Mean operative duration was 185.4 ± 54.0 min, and the mean intraoperative and postoperative blood loss volumes were 99.8 ± 120.8 mL and 366.5 ± 147.9 mL, respectively (Table 1). Two patients received red blood cell transfusion after the operation. None of the patients underwent reoperation because of postoperative bleeding or epidural hematoma. To exclude the effect of treatment with antithrombotic agents before surgery, we compared the volume of blood loss between patients taking antithrombotic drugs (n = 14) and those not taking antithrombotics (n = 90). There was no significant difference in perioperative bleeding between groups (S1 Table). We assessed the correlations between patient characteristics (other than the biological makers for hemostasis) and bleeding volume (Table 2). Intraoperative bleeding volume was significantly associated with operative duration, number of enlarged laminae, and alanine aminotransferase (ALT), while postoperative bleeding volume was significantly correlated with male sex, body weight, body mass index (BMI), the number of enlarged laminae, operative duration, and ALT (Table 2).

**Table 2:** Correlations of patient variables with perioperative bleeding*.

Variable	Intraoperative bleeding	Postoperative bleeding
Age	0.047	-0.114
Male gender	0.186	0.326‡
Body weight	0.120	0.414‡
BMI	0.071	0.304‡
Antithrombotic medications	0.018	0.046
Comorbidity		
Hypertension	-0.043	-0.064
Diabetes mellitus	0.184	0.056
Rheumatoid arthritis	0.002	-0.153
Hemodialysis	-0.066	-0.083
No. of enlarged laminae	0.238†	0.380‡
Operation time	0.291‡	0.309‡
White blood cell	0.023	-0.048
Red blood cell	-0.157	0.063
Hemoglobin	-0.096	0.129
AST	0.140	0.259
ALT	0.273‡	0.321‡
Blood urea nitrogen	0.103	-0.015
Creatinine	-0.040	0.015
CRP	-0.042	-0.178
ESR	0.044	-0.073
Primary hemostasis		
Platelet count	-0.287‡	-0.344‡
Platelet coagulation		
ADP-induced (2 μM)	0.065	-0.143
ADP-induced (5 μM)	-0.199†	-0.286‡
Collagen-induced (1 μg/ml)	-0.224†	-0.293‡
Collagen-induced (3 μg/ml)	-0.224†	-0.350‡
PAR-induced	-0.135	-0.198†
Blood coagulation		
%PT	-0.202†	-0.256‡
APTT	-0.045	-0.051
Antithrombin	0.144	-0.165
Soluble fibrin	-0.133	-0.231†
Fibrinolysis		
PAI-1	-0.202†	0.116
D-dimer	0.069	-0.119
Plasminogen	-0.019	-0.155
α2-PI	-0.038	-0.194†
PIC	0.116	-0.204†
Endothelial cell marker		
E-selectin	-0.017	-0.036

*Values are Pearson's correlation coefficients. BMI denotes Body-mass index; AST, aspartate transaminase; ALT, alanine aminotransferase; CRP, C-reactive protein; ESR, erythrocyte sedimentation rate; ADP, adenosine diphosphate; PAR, protease-activated receptor agonist; %PT, %prothrombin time; APTT, activated thromboplastin time; PAI-1, plasminogen activator inhibitor-1; α2-PI, α2-plasmin inhibitor; PIC, plasmin-α2-plasmin inhibitor complex.

†*P* < 0.05

‡*P* < 0.01.

### Role of primary hemostasis

**Fig 1:**
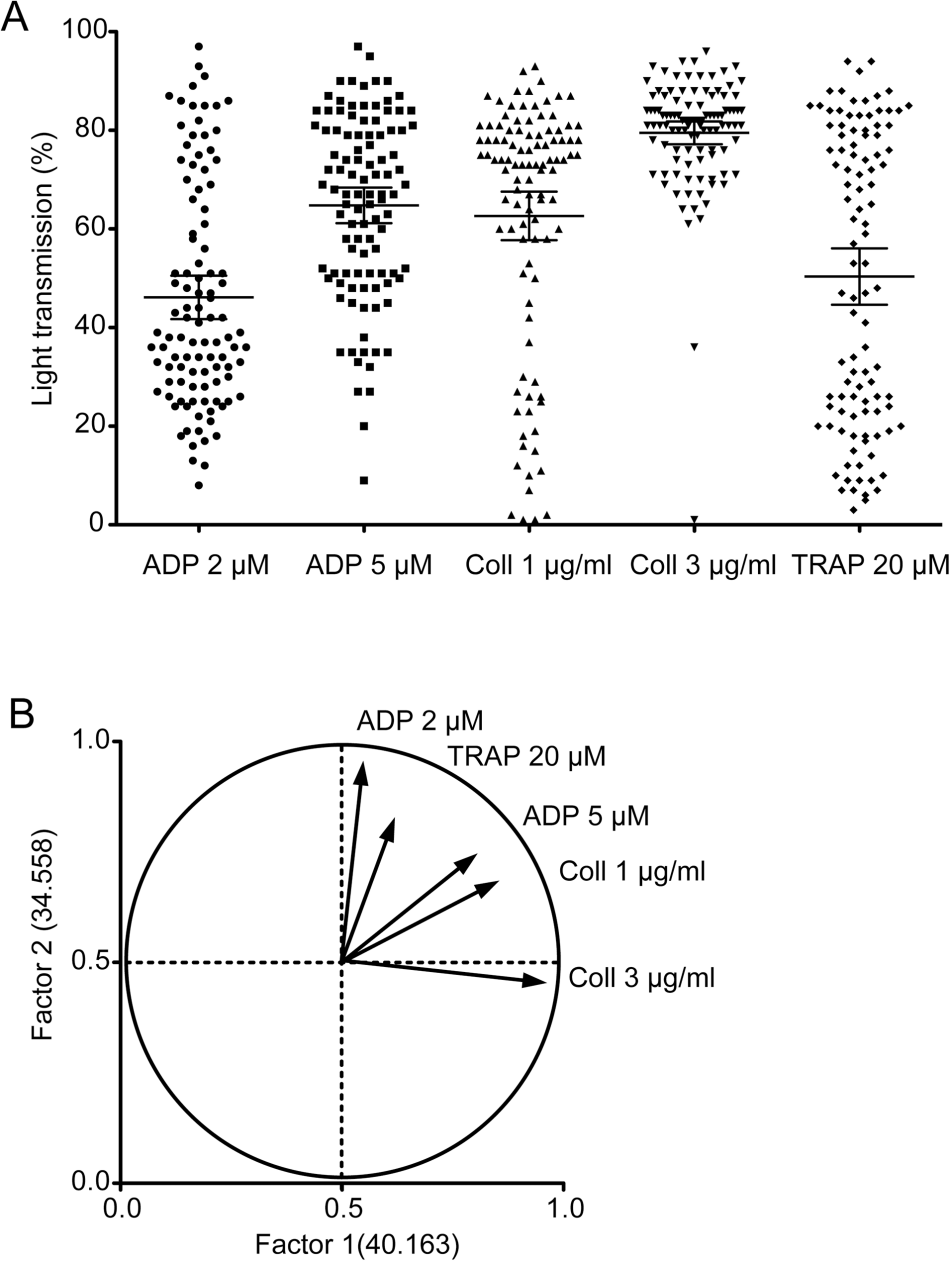
Strong correlation among platelet aggregations induced by different agonists. Platelet-rich plasma was stimulated with 2 μM ADP, 5 μM ADP, 1 μg/mL collagen, 3 μg/mL collagen, or 20 μM thrombin-receptor activating peptide (TRAP). Platelet aggregation was assessed by light transmission. Bars are means ± SEM. (A) Maximal light transmissions obtained from each examination are shown. (B) Factor analysis to identify latent factor to determine individual difference of platelet aggregation. Two latent factors (Factor 1, horizontal axis; Factor 2, vertical axis) explain the individual aggregation patterns stimulated with an indicated agonist.

To examine the role of primary hemostasis for perioperative bleeding, we employed light transmission aggregometry, a gold standard of platelet function testing (Fig 1A). Although bleeding time was mainly used as the conventional test of primary hemostasis, it has been thought to be of low specificity and sensitivity to evaluate platelet function [12]. There were significant correlations between the results of platelet aggregation induced by different agonists (ADP, collagen, and TRAP) within a given subject (data not shown). Factor analysis revealed that only two latent factors could explain 74.721% of individual difference of platelet aggregation (Fig 1B). In addition, the results of the platelet aggregation test were associated with bleeding volume (Table 2). In particular, collagen-induced platelet aggregation seemed to be the most important independent variable (Table 2). Not only platelet function assessed by aggregometry, but also platelet count, were associated with perioperative bleeding volume (Table 2).

### Coagulation factors and fibrinolysis

We next investigated the correlation of each factor with secondary hemostasis, fibrinolysis, and endothelial function (Table 3). There was a significant association among AT, prothrombin time (PT), α2-PI, and plasminogen. These results may be partly influenced by hepatic function, because these factors are synthesized in liver. PIC was correlated with D-dimer and SF, suggesting that increases in these factors can detect a prothrombotic state (Table 3). It was possible that increase in PAI-1 inhibited fibrinolysis because of negative correlation between PAI-1 and PIC (or D-dimer) (Table 3). In addition, the proteins synthesized from endothelial cells (PAI-1 and E-selectin) correlated with each other (Table 3). We further examined the correlations between laboratory data and perioperative bleeding volume (Table 2). Intraoperative blood loss was negatively associated with %PT and PAI-1 (Table 2). There were also negative correlations of postoperative blood loss with %PT, PIC, and α2-PI (Table 2). It is possible that decreased levels of PAI-1 and α2PI may accelerate surgical bleeding through the activation of fibrinolysis.

**Table 3:** Correlation between the markers for coagulation, fibrinolysis, and endothelial function*.

	APTT	%PT	PAI-1	D-dimer	ES	AT	α2-PI	Plg	PIC	SF
APTT	1	-0.285‡	-0.088	-0.062	-0.069	-0.213	-0.142	-0.114	0.174	0.471‡
PT%	-	1	-0.077	-0.160	-0.116	0.209‡	0.222†	0.289‡	-0.120	-0.133
PAI-1	-	-	1	-0.113‡	0.210†	-0.180	0.106	-0.0607	-0.369‡	-0.0559
D-dimer	-	-	-	1	0.005	0.007	-0.039	-0.095	0.354	0.235
ES	-	-	-	-	1	0.054	0.100	-0.044	-0.105	0.089
AT	-	-	-	-	-	1	0.474‡	0.503‡	0.109	0.064
α2-PI	-	-	-	-	-	-	1	0.447‡	0.039	0.103
Plg	-	-	-	-	-	-	-	1	0.310‡	0.099
PIC	-	-	-	-	-	-	-	-	1	0.360‡
SF	-	-	-	-	-	-	-	-	-	1

*Values are Pearson's correlation coefficients. APTT, activated thromboplastin time; %PT, %prothrombin time; PAI-1, plasminogen activator inhibitor-1; ES, E-selectin; AT, antithrombin; α2-PI, α2-plasmin inhibitor; Plg, plasminogen; PIC, plasmin-α2-plasmin inhibitor complex; SF, soluble fibrin monomer complex.

†*P* < 0.05

‡*P* < 0.01.

### Importance of each independent factor for bleeding volume

To compare the importance of each independent risk factor for perioperative bleeding, we performed multivariate regression analysis (Table 4). For intraoperative bleeding, operation time, PAI-1, ALT, and collagen-induced platelet aggregation independently predicted bleeding volume (Table 4). Of note, PAI-1 is the most important factor to determine the intraoperative bleeding volume among the variables. On the other hand, body weight was the most important predictor in determining postoperative bleeding volume (Table 4). The hemostatic tests including α2-PI, platelet count, and collagen-induced platelet aggregation were also associated with postoperative bleeding volume (Table 4). These data suggest that hemostatic functions (fibrinolysis inhibitors and platelet function) that could not be assessed by routine examination determine perioperative bleeding volume.

**Table 4:** Multiple regression analysis of factors associated with intra- and postoperative bleeding volume*.

Independent variable	Non-standardized coefficient β	95% CI for β	Standardized coefficient β	*P* value
Intraoperative bleeding				
Operation time	0.717	0.337 to 1.097	0.331	< 0.001
PAI-1	-3.004	-4.536 to -1.472	-0.348	< 0.001
ALT	1.726	0.635 to 2.816	0.281	0.002
Collagen-induced PA	-1.087	-1.885 to -0.289	-0.232	0.008
*R*^2^ = 0.263				
Postoperative independent variable	Non-standardized coefficient β	95% CI for β	Standardized coefficient β	*P* value
Body weight	3.884	2.197 to 5.492	0.362	< 0.001
Platelet count	-5.258	-9.034 to -1.483	-0.221	0.007
No. of enlarged laminae	86.026	37.698 to 134.354	0.281	0.001
α2-PI	-1.932	-3.397 to -0.467	-0.206	0.010
Collagen-induced PA	-2.218	-4.204 to -0.232	-0.178	0.029
*R*^2^ = 0.413				

*PAI-1, plasminogen activator inhibitor-1; ALT, alanine aminotransferase; Collagen-induced PA, 1 μg/ml collagen-induced platelet aggregation; α2-PI, α2-plasmin inhibitor.

### Association between body weight and PAI-1

**Fig 2:**
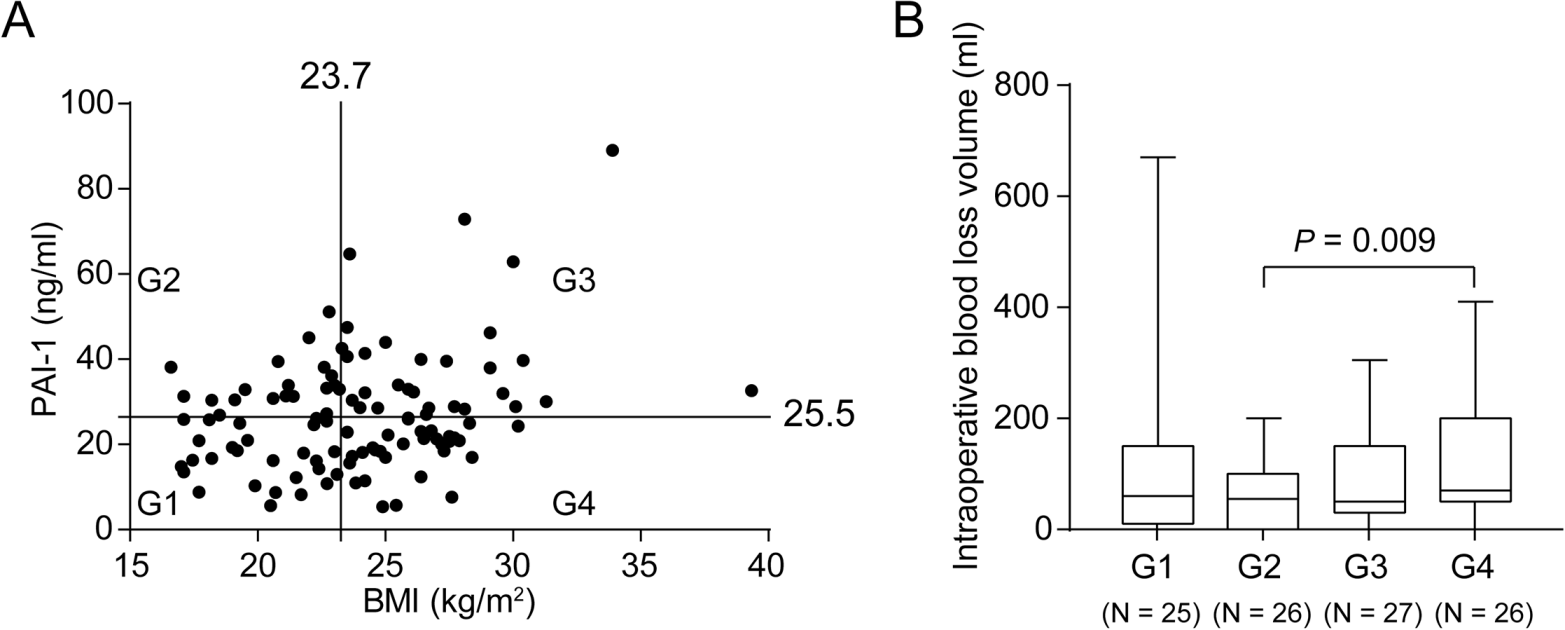
Low level of PAI-1 and obesity synergistically increase intraoperative bleeding. (A) Plasma PAI-1 level is significantly associated with BMI (*r* = 0.294, *P* = 0.002, n = 104). Patients are categorized into four groups by a median split of each value. (B) Blood-loss volume for each category is expressed as a box-and-whisker plot. The bottom and top of the box are the first and third quartiles, and the horizontal line inside the box represents the median. The ends of the whiskers represent the minimum and maximum values for all data. Statistical significance is observed between group 2 and group 4. BMI, body mass index; PAI-1, plasminogen activator inhibitor-1.

We finally investigated the underlying mechanism to determine the levels of PAI-1. There were significant positive correlations between PAI-1 and body weight (*r* = 0.2987, *P* = 0.002) and between PAI-1 concentration and BMI (*r =* 0.3031, *P* = 0.0017), as described previously [13]. Although the previous study showed a correlation between lipid profile and PAI-1 [14], we found no significant correlation between PAI-1 and total cholesterol levels (*r* = −0.038, *P* = 0.698). To examine the synergistic effect of low PAI-1 and obesity on intraoperative bleeding volume, we categorized the parent population into four groups (group 1, low BMI and low PAI-1; group 2, high PAI-1 and low BMI; group 3, high PAI-1 and high BMI; group 4, low PAI-1 and high BMI) (Fig 2A). The results from group 4 indicated a significant increase in blood loss compared with group 2 (Fig 2B), suggesting that obesity patients with low PAI-1 level have higher risk for perioperative bleeding.

## Discussion

Hemostasis is a physiological process that terminates bleeding after vascular injury to keep blood within vessels. Two basic mechanisms are required to efficiently promote a hemostatic plug, comprising platelets and the coagulation system [15]. Fibrinolysis also plays an important role in hemostasis. Pathological thrombosis and bleeding can occur through dysfunction of these mechanisms. While the differential roles of platelets and blood coagulation in regulating pathological thrombosis are well known [16], the temporal and specific roles of these components to terminate bleeding after surgery has not been elucidated. Here, we found that individual differences of hemostatic function determine posttraumatic bleeding volume even if patients lack a history of bleeding diathesis. Among several stages of hemostasis, our findings suggest the importance of regulating fibrinolytic pathway and platelet aggregability.

The most interesting result obtained in this study is the importance of fibrinolysis for perioperative hemostasis. A low level of PAI-1 was the most important determinant in predicting intraoperative bleeding volume. Fibrinolytic activation occurs in the majority of trauma patients, and the level of activation correlates with poor clinical outcome [17]. It is possible that low levels of PAI-1 resulted in unexpected activation of fibrinolysis during surgery to increase bleeding volume. Plasma levels of PAI-1 are in part genetically determined, and the importance of promoter gene 4G/5G polymorphism has been identified [18]. Furthermore, it is well known that expression of PAI-1 is easily upregulated by inflammatory cytokines including tumor necrosis factor alpha, transforming growth factor beta, and interleukins [19]. The increase in PAI-1 in response to acute inflammation contributes to the formation of fibrinolysis-resistant thrombosis in acute illness, while it may have a role as host defense mechanism to effectively terminate bleeding during surgery. An increase in PAI-1 in response to surgery reportedly showed a rapidly reverting acute-phase pattern [20]. We presume that the increase in PAI-1 acts as a principal regulator of fibrinolysis soon after the procedure (during the operation), because PAI-1 was quickly elevated by the surgical procedure. α2-PI may become important in determining fibrinolysis in postoperative bleeding, when the levels of PAI-1 return to basal levels after the surgical invasion. Rare bleeding diathesis in PAI-1 and α2-PI deficiencies in humans produced life-threatening spontaneous hemorrhage and prolonged wound healing [21, 22]. These rare deficiencies were successfully treated with tranexamic acid, an anti-fibrinolytic drug [23]. Targeting fibrinolysis may provide an important strategy to maintain hemostasis in the surgical setting of patients with low PAI-1 or α2-PI.

Our data suggest that body weight is the most important factor in determining postoperative bleeding volume. Obesity has become more common among patients considering orthopedic surgery, and previous meta-analysis revealed that obesity is associated with a higher risk of infection, bleeding, and longer surgical time [24]. It is clearly evident that anatomical problems in obese patients, e.g., thicker subcutaneous tissue and vessels in adipose tissue, lead to difficulty in achieving hemostasis as well as longer operation times; indeed, operation time was significantly correlated with body weight in our study (*r* = 0.2437, *P* = 0.0127). Furthermore, other factors may increase bleeding after surgery in obese patients. We found that there were significant negative correlations between body weight and BMI, and most of the platelet aggregation results, e.g., collagen-induced platelet aggregation, were negatively correlated with body weight (*r* = −0.2593, *P* = 0.0076) and BMI (*r =* −0.2012, *P* = 0.0395). It is possible that an increase in postoperative blood loss in obese patients is partly mediated by impairment of primary hemostasis due to platelet dysfunction.

Obese patients are also known to be at higher risk for thromboembolism. We should discriminate individuals at higher risk of bleeding from obese patients before surgery. The individualized therapy targeting obese patients seems to be particularly important in patients undergoing total hip arthroplasty and total knee arthroplasty, where antithrombotic prophylaxis is recommended to prevent venous thromboembolism. Increasing levels of PAI-1 were reportedly useful in distinguishing patients who develop venous thromboembolism after total hip arthroplasty [25] and is generally associated with the risk of thrombotic events during other surgical procedures [26–28]. Since our data suggest that obese patients with low PAI-1 levels may be at significant risk of perioperative bleeding, it is possible to safely manage patients with low-PAI-1 without antithrombotic drugs during surgical procedures. The assessment of PAI-1 may become a gatekeeper to distinguish hemorrhagic and thrombotic patients from obese patients.

There are several limitations to this study. First, the predictive models may have been underpowered by the relatively small sample size. Our models may have missed important associations or may have erroneously detected associations that are not clinically relevant. Second, the current results may not be equally applicable to other types of major surgery because cervical laminoplasty is associated with relatively small blood loss and short operation time. In addition, we measured only preoperative samples in this study. The measurement of intra- or postoperative blood samples more closely predicts the perioperative bleeding in the indicated period. We also could not define the cut-off value to make a decision for clinical use. We defined massive bleeding as the first quartile (145 ml for intraoperative bleeding; 645 ml for postoperative bleeding), and performed receiver operating characteristic (ROC) curve analysis. We found that the threshold of PAI-1 for intraoperative bleeding was 30.4 ng/ml (area under the curve (AUC) = 0.655, sensitivity = 0.846, 1 − specificity = 0.575) and body weight for postoperative bleeding was 65.0 kg (AUC = 0.658, sensitivity = 0.692, 1 − specificity = 0.354). However, the value of AUC showed low accuracy in predicting bleeding, and we could not define clinically important bleeding because only two patients received red blood cell transfusions. Further large-scale prospective studies are required to determine which markers are associated with the risk of clinically important bleeding after surgery, to set the cut-point of each marker for prediction of clinically relevant bleeding, and to establish safer approaches to administering antithrombotic drugs during surgical procedures by measuring detailed hemostatic markers.

In conclusion, our data demonstrate that the inter-individual viability of fibrinolytic pathway and platelet aggregability determines perioperative bleeding in patients without a history of bleeding. Measurement of PAI-1, α2-PI, and platelet aggregation, in addition to the current routine laboratory tests, may improve the ability to predict patients with an increased risk for excessive bleeding during surgery. This information could be used to determine which patients require additional interventions, including antifibrinolytic treatment, and thereby increase the safety associated with these treatments. Larger prospective studies are required to determine which biological markers are beneficial to identify bleeding risk in the future.

## Supporting information

S1 Table Comparison between patients with or without antithrombotic therapy. (PDF)
