# Supplementary material for: Hemostatic function to regulate perioperative bleeding in patients undergoing spinal surgery: A prospective observational study
Source: PLoS One. 2017 Jun 16;12(6):e0179829. doi: 10.1371/journal.pone.0179829 (PMC5473574; doi:10.1371/journal.pone.0179829)
Supplement: S1 Table — (PDF) [file pone.0179829.s001.pdf]

S1 Table Comparison between Patients with or without Antithrombotic Therapy

Variable	Patients with antithrombotic medication (n = 14)	Patients without antithrombotic medication (n = 90)	*P* value
Age (year)	72.8 ± 8.0	64.4 ± 10.5	0.005†
BMI	23.4 ± 3.4	24.8 ± 3.7	0.737
Intraoperative bleeding (ml)	104.3 ± 98.8	99.1 ± 124.3	0.883
Postoperative bleeding (ml)	383.6 ± 111.9	363.8 ± 153.1	0.645
ALT	15.9 ± 5.7	25.2 ± 20.3	0.091
BUN	17.0 ± 2.0	15.8 ± 7.2	0.591
creatine	1.7 ± 2.8	0.9 ± 0.1	0.035†
Platelet count	19.6 ± 5.5	22.3 ± 6.4	0.142
%PT	89.5 ± 15.1	99.9 ± 12.7	0.007†
APTT	37.9 ± 13.1	31.1 ± 3.1	<0.001†
PAI-1	26.2 ± 11.3	6.7 ± 14.2	0.898
Plasminogen	89.6 ± 21.1	93.9 ± 16.1	0.370
α2-PI	84.8 ± 16.1	88.0 ± 15.7	0.477
FDP	5.0 ± 1.7	4.9 ± 6.0	0.991
Antithrombin	78.3 ± 20.4	89.9 ± 15.4	0.015†
PPI	1.0 ± 0.4	0.9 ± 0.3	0.503
Platelet aggregation			
ADP-induced	65.0 ± 14.3	63.7 ± 19.9	0.637
Collagen-induced	77.3 ± 13.6	78.9 ± 14.3	0.686
PAR-induced	59.9 ± 24.1	49.4 ± 30.9	0.225

*Values are reported as mean ± standard deviation. †Statistically significant ($P < 0.05$), unpaired *t*-test. BMI denotes Body-mass index; ALT, alanine aminotransferase; %PT, %prothrombin time; APTT, activated thromboplastin time; PAI-1, plasminogen activator inhibitor-1; α2-PI, α2-plasmin inhibitor; FDP, fibrin degradation products; PPI, plasmin-α2-plasmin inhibitor complex; ADP, adenosine diphosphate; PAR, protease-activated receptor agonist.
